# The Consequences of Chromosome Segregation Errors in Mitosis and Meiosis

**DOI:** 10.3390/biology6010012

**Published:** 2017-02-08

**Authors:** Tamara Potapova, Gary J. Gorbsky

**Affiliations:** 1 Stowers Institute for Medical Research, Kansas City, MO 64110, USA; tpo@stowers.org; 2 Cell Cycle and Cancer Biology Research Program, Oklahoma Medical Research Foundation, Oklahoma City, OK 73104, USA

**Keywords:** aneuploidy, polyploidy, microtubule, chromosome instability, cancer, birth defects, fertility, drug resistance, centromere, kinetochore

## Abstract

Mistakes during cell division frequently generate changes in chromosome content, producing aneuploid or polyploid progeny cells. Polyploid cells may then undergo abnormal division to generate aneuploid cells. Chromosome segregation errors may also involve fragments of whole chromosomes. A major consequence of segregation defects is change in the relative dosage of products from genes located on the missegregated chromosomes. Abnormal expression of transcriptional regulators can also impact genes on the properly segregated chromosomes. The consequences of these perturbations in gene expression depend on the specific chromosomes affected and on the interplay of the aneuploid phenotype with the environment. Most often, these novel chromosome distributions are detrimental to the health and survival of the organism. However, in a changed environment, alterations in gene copy number may generate a more highly adapted phenotype. Chromosome segregation errors also have important implications in human health. They may promote drug resistance in pathogenic microorganisms. In cancer cells, they are a source for genetic and phenotypic variability that may select for populations with increased malignance and resistance to therapy. Lastly, chromosome segregation errors during gamete formation in meiosis are a primary cause of human birth defects and infertility. This review describes the consequences of mitotic and meiotic errors focusing on novel concepts and human health.

## 1. Introduction

**Figure 1:**
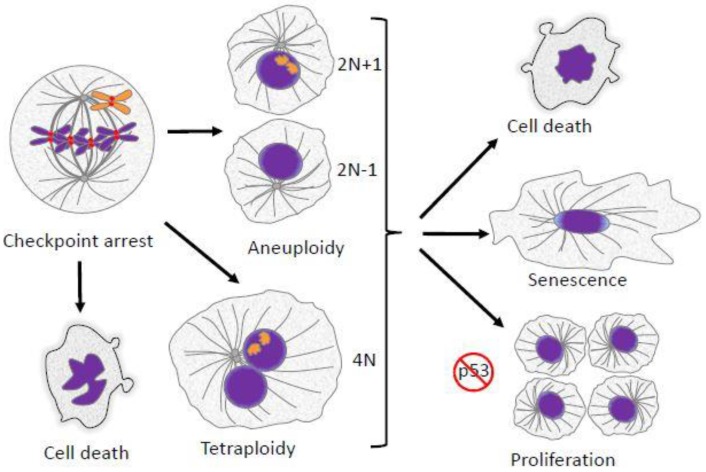
Mitotic defects have several potential outcomes. Failed alignment of chromosomes leads to mitotic arrest/delay enforced by the spindle checkpoint. If the failed alignment is not corrected, cells can follow several fates. They can undergo cell death directly from mitotic arrest. Cells may also suffer various kinds of abnormalities during mitotic exit, leading to the formation of aneuploid progeny. Alternatively, cells may exit mitosis without proper chromosome segregation and cytokinesis, resulting in a formation of a single tetraploid cell. Aneuploid or polyploid daughter cells may undergo cell death, cessation of proliferation and senescence, or continued proliferation. In most cases continued proliferation requires suppression or inactivation of the p53 tumor suppressor pathway.

Other papers in the Special Issue “Mechanisms of Mitotic Chromosome Segregation” have explored the events of cell division and how defects might generate errors in the transmission of chromosomes to progeny cells. The defects are diverse in origin, including abnormalities in chromosome structure and function resulting in chromosomes that lag in anaphase or exhibit incomplete separation of sister chromatids. Spindle abnormalities such as multipolar spindles and defects in cytokinesis are additional sources of abnormal chromosome segregation. Finally, errors in cell cycle regulation, including delays during division and defects in cell cycle checkpoints also lead to missegregation. In this concluding chapter, we delve into the consequences of mitotic and meiotic errors. These can be benign or severe, depending on the degree and nature of the error, on the genetic background of the cell, and on the precise role of the cell in question. It is important to recognize that segregation abnormalities may not always generate aneuploidy. Even for a single chromosome that undergoes premature chromatid separation, random assortment will generate proper segregation to the two daughter cells 50% of the time. Outcomes of improper segregation are also influenced by stochastic variables that cause cells in seemingly identical situations to take different paths in response to identical errors [1]. Cell cycle checkpoints can sometimes identify impending errors and provide corrective countermeasures that lead to normal division. If checkpoints fail to correct the problem but division proceeds, then daughter cells are born with a genetic imbalance of one or more whole chromosomes, segments of chromosomes, or entire sets of chromosomes. In some instances, departure from conventional cell cycle patterns that lead to abnormal chromosome content are an aspect of normal development. This is often true for polyploidy, while aneuploidy is more often a result of errors in chromosome segregation. In most normal tissue cells, a surveillance system, highly dependent on the p53 tumor suppressor, is active in responding to the presence of abnormal chromosome content and can halt the cycling of the cell, cause cell death, or induce senescence (Figure 1) [2,3,4,5].

Cells that lose or gain less than a whole set of chromosomes during cell division are termed aneuploid. Cells with a tendency to lose or gain chromosomes at a high rate are said to exhibit chromosome instability (see Table 1 for definitions). Certain genotypes may be inherently prone to continuous chromosomal instability, producing a diverse brood of aneuploid progeny. Alternatively, cells can be aneuploid but relatively stable in chromosome content. Cells may also undergo an increase in a whole set of chromosomes, a condition termed polyploidy. Polyploid cells frequently contain more than two centrosomes. In subsequent cell divisions, the centrosomes sometimes generate multipolar spindles where chromosomes are segregated to three or more daughter cells, resulting in aneuploid cells with variable numbers of chromosomes. The full consequences of chromosome segregation errors are vast in scope, since they affect many aspects of cell physiology, tissue homeostasis, and the adaptability of cells and organisms.

Chromosome segregation requires the coordination of two major pathways: chromosome movement and cell cycle regulation during M phase. A major contributor to this coordination is the mitotic spindle checkpoint. As detailed in another contribution in this series, defects in mitotic spindle assembly and chromosome alignment activate the spindle checkpoint, which delays cells in M phase. Optimally this delay allows the recovery of the normal spindle and balanced chromosome segregation. However, the delay can have multiple consequences. Mammalian cells arrested in M phase eventually exhibit markers indicative of DNA damage [6,7]. Cells in which the spindle checkpoint is purposefully activated by application of microtubule drugs often undergo apoptotic cell death, either directly in mitosis or after exiting M phase into G1 (Figure 1) [1,8]. One critical pathway in regulating cell death during M phase arrest is Cdk1-dependent phosphorylation and the subsequent degradation of Mcl-1, an anti-apoptotic member of the Bcl-2 family [9,10,11,12]. Certain aspects of apoptotic signaling are suppressed during M phase, but partial activation of these pathways may lead to cell death in the subsequent G1 phase [13]. In cells with normal p53 function, even a relatively short delay in M phase may lead cells to cease cycling after entering G1 [14]. Cells with chromosome segregation defects that escape apoptosis produce progeny with altered chromosome content. These cells may continue to cycle, particularly if p53 is inactivated. Chromosome segregation errors result in aneuploid or polyploid cells and are generally detrimental to both the cell and the organism. However, in some instances, changes in ploidy are programmed in normal development and physiology. At times, even accidental diversions from euploidy can generate beneficial evolutionary adaptations, particularly in single-cell organisms. In this review, we describe the consequences of aneuploidy and polyploidy due to segregation errors in mitosis and meiosis, focusing on recent novel ideas and on topics pertinent to human health.

## 2. Aneuploidy in Mitosis

### 2.1. Effects of Aneuploidy on Gene Dosage

In diploid organisms, apart from special instances such as the sex chromosomes of animals, genes are present in two copies that are both transcribed. Gain or loss of one copy changes the amount of gene product produced, a property called gene dosage. Unlike the doubling of a whole genome in polyploidy, where the increase of the gene dosage is equivalent for all chromosomes, loss or gain of an individual chromosome or chromosome fragment causes unbalanced changes in the cellular proteome. Studies in fungi and mammalian systems have shown that changes in mRNA and protein levels from genes on aneuploid chromosomes are roughly proportional to the changes in the chromosome copy number [15,16,17,18,19]. Aneuploidy of large, gene-rich chromosomes can cause changes in the expression of thousands of genes. Moreover, transcription factors encoded on aneuploid chromosomes will alter gene expression on other chromosomes [19]. Consequently, aneuploidy can cause a diverse spectrum of changes in the proteome of the cell, depending on the specific chromosomes lost or gained. Finally, aneuploidy itself may drive further chromosome instability, a topic treated in more detail below.

### 2.2. Effects of Aneuploidy on Cellular Fitness

**Figure 2:**
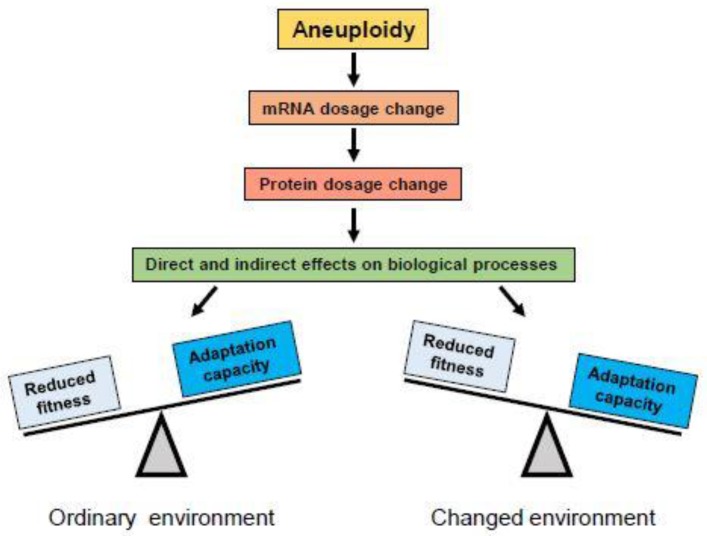
Aneuploidy produces changes in mRNA dosage which lead to changes in protein dosage for genes on the gained or lost chromosome(s). Changed protein levels can have direct effects on biological processes in which they are involved, or change the stoichiometry of protein complexes of which they are components, causing changes in their function. Changes in gene dosage of regulatory proteins like transcription factors may also exert indirect effects on biological processes by altering expression of their target genes on other chromosomes. In most cases, alterations in protein levels are disadvantageous or detrimental for organisms adapted to their ordinary environment (left), where the euploid karyotype provides best fitness. However, under conditions of environmental change, rapid alterations in expression of many genes may provide adaptive potential and be selected (right).

The euploid karyotype is a product of natural selection for the best fitness for a species in an ecological niche. Aneuploidy will alter cellular physiology in many ways, depending on which chromosomes are extra or missing. Changes in the chromosome copy number alter the production of proteins (Figure 2). Imbalances in protein levels will occur particularly in protein complexes where the genes encoding individual components of the complex reside on different chromosomes. These imbalances will disrupt protein homeostasis, increase the workload for chaperones, and overload the protein degradation machinery, resulting in a form of toxicity referred to as proteotoxic stress [18,20]. Therefore, aneuploidy imposes a distinct fitness cost. In the ordinary environment, engineered aneuploid yeast strains and viable aneuploid mouse cells often grow slower than their euploid counterparts [21,22,23].

### 2.3. Aneuploidy in Fungi

Despite the overall fitness cost of carrying extra chromosomes, variation in the chromosome copy number can be found in many fungal species. The immediate shifts in the dosage of several genes conveyed by aneuploidy may confer rapid advantages in new environments, compared to the slower method via selection for adaptive mutations in individual genes. Fungi display remarkable genomic plasticity and can tolerate large-scale genomic changes. Variations in the chromosome copy number in *S. cerevisiae* have been detected frequently in association with domestication and adaptation to specific, often suboptimal, environments [24,25,26,27,28]. Aneuploidies that have deleterious phenotypes are quickly eliminated from populations by selection, leaving viable aneuploidies where the benefits of the presence of extra chromosome(s) outweigh the fitness cost (Figure 2). For instance, aneuploidy is common in laboratory strains of *S. cerevisiae* exposed to genetic transformation techniques, and in wild strains from diverse natural environments [29,30]. It was estimated that in the laboratory deletion collection of *S. cerevisiae* mutant strains, approximately 8% are aneuploid [31]. Aneuploidy appears to be more common in diploid versus haploid strains [32], consistent with the idea that smaller gene dosage changes are more tolerable. In industrial strains of *S. cerevisiae* cultured in fermenters or bioreactors, whole chromosome aneuploidies have repeatedly emerged in response to suboptimal conditions such as glucose or phosphate stress [26,32]. Therefore, the presence and frequency of aneuploidy appears to be strongly influenced by the environment. Karyotypic abnormalities are also frequently found in hospital isolates of pathogenic fungi *Candida albicans* and *Cryptococcus neoformans*, and aneuploidy in these pathogens has been associated with increased virulence and drug resistance [33,34,35,36,37].

### 2.4. Aneuploidy in Mammalian Cells

Aneuploidies commonly emerge in mammalian cell cultures, but this phenomenon has been traditionally viewed as an annoyance rather than a topic worthy of study. Most tissue culture cell lines are highly aneuploid, in part because many were originally derived from aneuploid tumors, but also because of fluctuations in ploidy with continued culture. Recently, the de novo generation of aneuploidy in cultured cells has emerged as an important concern for the therapeutic use of pluripotent human stem cells, where the correct karyotype is essential. Pluripotent stem cells acquire numerical and structural chromosomal aberrations during prolonged cell culture, and concurrently show initial signs of malignant transformation [38,39,40]. Human stem cell lines frequently gain a copy of chromosome 12. Trisomy 12 increases cell proliferation in culture and corresponds to global changes in the transcriptome, making the gene expression profile of aneuploid stem cells similar to germ cell tumors [38]. While gaining an extra copy of chromosome 12 allows trisomic stem cells to thrive in tissue culture conditions, it has obvious detrimental consequences for the therapeutic use of these cells. A recent study generated a panel of trisomic mouse embryonic stem cells lines, each carrying an extra copy of single chromosomes 6, 8, 11, 12, or 15. Most of these trisomic cell lines proliferated at a high rate in culture, but showed a reduced ability to differentiate and an increased potential to form teratomas, possibly due to deregulated gene expression [41].

Retinal Pigment Epithelium-1 (RPE-1) cells are a diploid line of human retinal pigment epithelium cells that are engineered to express telomerase, rendering them immortal [42]. They are widely used as a model of non-malignant human diploid cells. In continuous passage in culture, RPE-1 cells will spontaneously gain an extra copy of chromosome 12 [43,44], but for a different reason than pluripotent stem cells. One of the copies of chromosome 12 in this cell line carries a mutation in the RAS gene [45], which may provide these cells a growth advantage. A study of immortalized human colonic epithelial cells carrying an extra copy of chromosome 7 showed better growth in serum-free medium compared to the isogenic diploid cells [46]. The growth advantage of the trisomic cells was postulated to stem from overexpression of the epidermal growth factor receptor gene located on chromosome 7. Aneuploid cells derived from the colorectal cancer cell line DLD1 engineered to be trisomic for chromosomes 7 or 13 also demonstrated selective growth advantages under various suboptimal conditions, such as serum deprivation, hypoxia, or exposure to the chemotherapeutic agent 5-fluorouracil [47]. Thus, while aneuploidy may be deleterious under conditions where the euploid karyotype provides the best fitness, it can provide a selective advantage when conditions change, allowing adaptation to a novel environment.

### 2.5. Aneuploidy as a Driver for Genomic and Chromosomal Instability

Chromosomal instability refers to an increased propensity for chromosome segregation errors, resulting in aneuploidy and genomic imbalances [48,49]. Aneuploidy itself may foster chromosome instability by perturbing the stoichiometry of proteins involved in chromosome segregation [50,51]. Studies in aneuploid budding yeast showed that unbalanced changes in the copy number of chromosome VII and X perturbed the ratio of essential spindle checkpoint proteins Mad1 and Mad2, whose genes are located, respectively, on these two chromosomes. Maintaining the 1:1 stoichiometry of the Mad1:Mad2 ratio appears crucial for monitoring kinetochore attachments [52]. Changes in Mad1:Mad2 ratios compromised the spindle checkpoint and increased chromosomal instability [53]. Importantly, aneuploid strains that gained copies of chromosomes VII and X simultaneously were karyotypically more stable than strains where these chromosomes were gained individually. Changing stoichiometric ratios of components of protein complexes or individual proteins with essential structural or regulatory functions can have severe consequences. Dosage imbalance among components of protein complexes can induce defects in the maintenance of genomic fidelity, affecting mitosis, cytokinesis, DNA replication, DNA repair, etc. In line with this hypothesis, yeast strains aneuploid in different chromosomes show various degrees of karyotypic and genomic instability, likely due to products from genes encoded on the aneuploid chromosomes [53,54]. Studies in human cells also suggest that aneuploidy itself may increase chromosome instability by affecting chromosome segregation in mitosis or by inducing defects in replication [55,56].

### 2.6. Aneuploidy and Cancer

In the late 19th century, it was recognized that tumor cells often exhibit abnormal, asymmetric mitotic figures [57]. In the early 20th century, Boveri proposed that abnormal chromosome content was the source for tumor malignancy, the earliest molecular hypothesis for the origin of cancer [58]. It is estimated that approximately 86% of solid tumors and 72% of hematopoietic cancers exhibit aneuploidy [59]. The question of whether aneuploidy is a cause or consequence of cancer has generated considerable controversy [60,61]. In general, most cancers display various degrees of genomic instability, including point mutations, chromosomal rearrangements, and changes in whole chromosome ploidy [62]. High levels of genomic instability generally correlate with more aggressive tumors and poorer patient prognosis. The environment of cancers can be thought of as complex cellular ecosystems that are constantly evolving, responding to challenges such as depletion of oxygen and nutrients, immune assault, and medical attempts at therapy [63]. Cancer cells must adapt to challenges in their microenvironment, and aneuploidy serves as an enabling factor in tumor evolution. An example of the adaptive evolution of cancer cells via aneuploidy is the loss of the heterozygosity of tumor suppressor genes. Inactivating mutations occur in many tumor suppressor genes, such the Retinoblastoma gene (Rb), but a single wild-type copy maintains suppressor function. Loss of the chromosome containing the wild-type copy leads to the loss of tumor suppressor function in the cancer cells.

Mouse models have aided in testing the role of aneuploidy in the origin and progression of cancer. Either underexpression or overexpression of mitotic regulators fosters both aneuploidy and increased cancer predisposition. For example, mice engineered to underexpress or overexpress most components of the spindle checkpoint pathway exhibit aneuploidy and tissue-specific increases in cancer incidence [64,65,66,67,68]. Complete ablation of the *BUB1B* gene that encodes the BubR1 checkpoint protein is embryonically lethal, but hypomorphs show increased aneuploidy, increased susceptibility to carcinogen-induced tumors, and accelerated aging phenotypes [69,70,71,72,73]. Surprisingly, in contrast to the usual consequences of overexpression of spindle checkpoint proteins, overproduction of BubR1 protects against cancer and other aging phenotypes and extends lifespan [74,75]. In humans, a rare genetic disease called Mosaic Variegated Aneuploidy stems from mutations in the *BUB1B* gene, and afflicted individuals show a very high proportion of aneuploid tissue cells. These patients suffer from a variety of serious pathologies, including growth defects, microcephaly, and increased cancer incidence [76,77,78].

Mouse embryos, heterozygous for a deletion of the gene encoding the mitotic kinesin protein, Cenp-E, show a weakened spindle checkpoint, and their cells will often enter anaphase in the presence of one or a few unaligned chromosomes [79]. The animals develop normally but are more prone to developing certain types of spontaneous tumors, such as lymphomas in the spleen and pulmonary adenomas in the lung. However, they are partially protected from other cancers, such as liver tumors [79]. Thus, depending on the context, aneuploidy can promote or inhibit oncogenesis. Crossing Cenp-E heterozygotes with other mutants that further increase the rate of chromosome missegregation led to tumor suppression, suggesting that the amount of chromosome missegregation may be important, whereby low rates promote tumor growth and high rates suppress it [80].

The potential biphasic effect of chromosome missegregation, to promote tumorigenesis at low levels and inhibit tumorigenesis at high levels, may have significance for the use of anti-mitotic drugs in cancer therapy. Taxol, the common name for the drug paclitaxel, is one of the most widely prescribed anti-cancer drugs. It binds and hyperstabilizes microtubules both in the test tube and in cells [81,82]. In cell culture, at moderate concentrations, it arrests cells in mitosis by activation of the spindle checkpoint [83,84]. Thus, for many years, the common assumption was that mitotic arrest was the mechanism underlying Taxol’s effectiveness in cancer therapy. However, the relatively low mitotic index in tumors in humans compared with Taxol’s rapid ability to shrink some tumors led to proposals that Taxol’s medical effectiveness might stem from targeting interphase tumor cells or the tumor environment [85,86]. A combined clinical and cell culture study led to the proposal that Taxol kills tumor cells in patients, not by mitotic arrest, but by increasing the propensity of tumor cells to undergo multipolar mitosis, leading to massive chromosome missegregation and tumor cell death [87]. Thus, while low levels of chromosome missegregation may be dangerous in promoting cancer, therapeutically driving missegregation to very high levels may conversely be an effective anti-cancer strategy.

Human cancers also exhibit genome instability due to dysfunction of chromosome telomeres, which may become too short after multiple rounds of replication (telomere attrition), or lose structural features such as telomere caps due to enzymatic defects [88]. Telomere defects generate broken ends on chromosomes, which may then fuse to similar broken ends on other chromosomes and generate dicentric chromosomes, in which a single chromosome will contain two widely separated centromeres. After this dicentric chromosome replicates and condenses in mitosis, each chromatid will harbor two centromeres that may orient toward opposite poles. During anaphase such a chromatid will form a chromosome bridge. This bridge can be severed during anaphase and form new broken ends. This mechanism can propagate breakage-fusion-bridge cycles that lead to complex chromosome rearrangements characteristic of tumor cells [89].

### 2.7. Cohesion Fatigue and Centromere Fission

Another potential source of aneuploidy in tumor cells is cohesion fatigue in cells that are delayed or arrested at metaphase. Normally metaphase is transient, lasting only a few minutes, and is followed by the onset of anaphase where the protease, Separase, severs Rad21, a component of the cohesin complex that holds sister chromatids together [90]. However, even when most chromosomes are aligned at the spindle midplane, anaphase onset may be delayed by the spindle checkpoint, activated by the failure of one or a few chromosomes to align in a timely manner [91]. Alternatively, defects in the expression of mitotic regulators, sometimes as a consequence of oncogenic changes, may induce delays at metaphase [68,92,93,94,95,96]. During the delay, chromosomes at the metaphase plate begin to separate asynchronously due to pulling forces of kinetochores on spindle microtubules, a process termed cohesion fatigue [92,97,98,99]. Cohesion fatigue is a general phenomenon, independent of the mechanism used to induce the metaphase delay [97,98,99]. The timing of chromatid separation varies among cell types and among the individual chromosomes within a single cell. Separation initiates at the kinetochores and then spreads distally along the chromosome arms [97].

**Figure 3:**
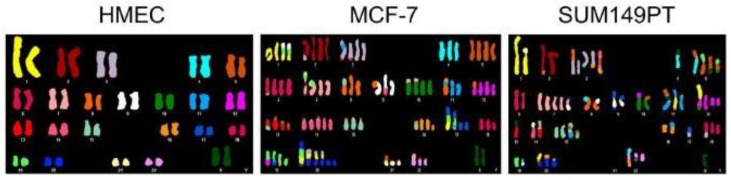
Cell lines derived from cancers exhibit numerical and segmental aneuploidy. Spectral karyotype comparison of normal human mammary epithelial cells (HMEC) and two breast cancer cell lines (MCF-7 and SUM149PT) that exhibit extensive numerical and segmental aneuploidy. Image reproduced from [106].

**Figure 4:**
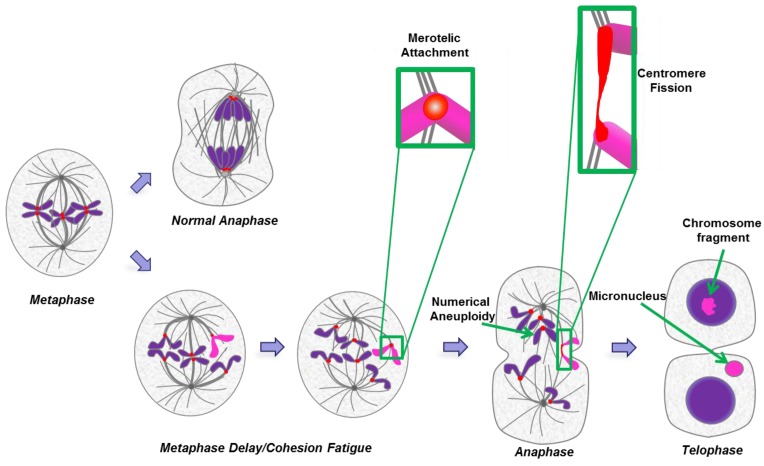
Numerical and segmental aneuploidy as an outcome of cohesion fatigue and centromere fission. A cell at metaphase will normally undergo balanced chromosome segregation in normal anaphase (upper path). If metaphase is delayed (lower path), chromatids may begin to undergo cohesion fatigue and separate. When sister chromatids separate they may both move to one of the two spindle poles leading to numerical aneuploidy following anaphase and mitotic exit. In other cases the kinetochore of an individual chromatid may undergo merotelic attachment to microtubules from both spindle poles (exemplified by sequential stages for the pink chromatid and detailed in the green boxes). Under this circumstance, spindle forces or cytokinesis may sever the chromatid resulting in chromosome fragments that can attach to other chromosomes resulting in segmental deletions, duplications, translocations and the formation of micronuclei.

In cancer cells, numerical and segmental aneuploidies are generally found together (Figure 3). Individually, many of the postulated origins of aneuploidy such as telomere erosion, DNA replication errors, DNA repair defects, or cytokinesis failure cannot in a simple way account for both numerical and structural chromosome abnormalities. As a result, many theories suggest independent origins for numerical and segmental aneuploidy. However, mitotic segregation defects can simultaneously generate both types of aneuploidy [100]. A common mitotic error is merotelic attachment whereby an individual kinetochore attaches to microtubules from both spindle poles. These attachment errors had been thought to be particularly dangerous because merotelic attachments are not well detected by the spindle checkpoint and allow progression to anaphase [101,102]. However, merotely that occurs during prometaphase may have a relatively minor effect on chromosome segregation because the merotelic kinetochore is more strongly attached to microtubules from the proper pole. Such merotelic chromatids are resolved properly in anaphase and do not result in chromosome missegregation [103,104]. In contrast, defects that occur after complete or partial chromatid separation in cohesion fatigue are likely to have severe consequences since the unattached kinetochore may be more prone to near equal attachment to both spindle poles. Kinetochores from partially or fully separated chromatids may attach to microtubules from both poles. Single chromatids derived from unreplicated DNA or from cohesion fatigue will sometimes congress to the spindle equator [101,105]. Alternatively, the two unpaired chromatids of a single chromosome may orient to the same pole, eventually resulting in numerical aneuploidy (Figure 4).

**Figure 5:**
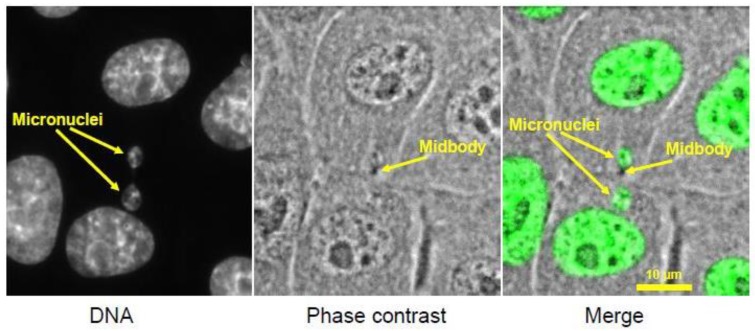
Micronuclei present in two daughter LLC-Pk cells, a cell line derived from porcine kidney. Micronuclei likely formed from lagging chromosomes that were partially trapped in the cytokinetic cleavage furrow. The midbody, the remnant of the cleavage furrow, bisects the region between the two micronuclei. (micrographs courtesy of Hem Sapkota.)

The study of cultured cells from mutant mice and analysis of human cancers of recent origin have led to a proposal that segmental aneuploidy defects involve whole chromosome arms that arise through “centromere fission”, breaks at or near centromeres [107,108]. Light and electron micrographs show that, under certain conditions, merotelically attached kinetochores undergo extreme stretching and possible severing [109]. Thus, merotelic attachment following partial or full chromatid separation in cohesion fatigue could generate both breaks at or near centromeres that give rise to duplications, deletions, and translocations as well as micronuclei (Figure 5). The potential consequences of micronuclei formation are described in detail below (Section 3). The fusion of broken arms containing centromeres to intact chromosomes may generate dicentric chromosomes that would then undergo rounds of breakage-fusion-bridge cycles to generate more complex and varied segmental chromosome defects. Cohesion fatigue in a large proportion of chromosomes, followed by subsequent cell division, is unlikely to generate viable daughter cells. However, partial or complete chromatid separation, occurring in just one or a few chromosomes, after shorter metaphase delays, may be a common initiating event for the numerical and segmental aneuploidies seen in cancer.

### 2.8. Modern Analysis and Implications of Cancer Aneuploidy

For many years, aneuploidy in tumors was studied using cytogenetic methods, which can accurately detect large karyotypic alterations but are less accurate in identifying small alterations. Comparative genomic hybridization and, more recently, next-generation sequencing techniques have enabled the detection of large and small copy number variations with higher resolution. Progress in sequencing technology has enabled large-scale cancer studies. The largest multi-institutional collaboration project The Cancer Genome Atlas (TCGA) has generated genomic data across many types of cancer and has shown that nearly all cancers have significant chromosome aberrations [110]. Chromosomal regions affected by copy number alterations in cancer vary widely in size. Among copy number variations, small (focal) amplifications or deletions are most frequent, followed by large-scale alterations: gain/loss of a chromosome arm or a whole chromosome. Interestingly, large-scale alterations typically show a low amplitude of amplification (i.e., gain or loss of one copy), but focal amplifications overall have a higher amplitude, indicating that small chromosomal segments can be duplicated multiple times [111].

Modern computational methods to analyze aneuploidy in multiple human tumor samples have generated a list of 70 genes highly correlated with chromosome instability, called the CIN70, and high expression of these genes correlates with adverse patient outcome [112,113]. Interestingly, a study of over 2000 breast cancer patients revealed a biphasic effect where the patients with the very highest CIN70 scores showed a better prognosis than those with intermediate scores [114]. On the other hand, for pathologists, frequent abnormal or atypical mitotic figures in human cancer tumors generally signify high malignancy. Aggressive cancers also exhibit abnormally shaped interphase nuclei and micronuclei that are post-mitotic footprints of severe mitotic problems [115]. In such cancers, the degree of karyotypic instability is likely to be very high.

Many copy number aberrations are unique, while others are recurrent. Recurrent copy number variations may indicate that alteration of this chromosomal segment functions as a driver in cancer progression. For instance, amplifications frequently encompass genomic regions that contain canonical oncogenes such as Cyclin D1, c-Myc, and ErbB-1. Deletions often include regions encoding canonical tumor suppressors such as *p16INK4A*/*p14ARF* and *PTEN*. Amplifications or deletions in these cases will result in corresponding changes in gene dosages of oncogenes or tumor suppressors, giving these karyotypes a selective proliferative advantage [116,117]. A recent computational analysis of the gene copy number in thousands of tumor-normal tissue pairs identified hundreds of novel potential oncogenes and tumor suppressor genes, many of which were correlated with whole chromosome and large segmental aneuploidies in the tumors [118]. However, verification of these guilt-by-association indications will require functional tests.

As a consequence of genomic instability, cells within a tumor can diverge and form distinct subpopulations, resulting in genomic heterogeneity within a tumor [119,120,121]. Clonal diversity is a prominent feature in many cancers, especially advanced ones, and is likely to play a role in tumor evolution and resistance to therapy [122]. Genomic studies of intra-tumor heterogeneity in a complex population of cells have been very challenging, but became possible with the development of single-cell sequencing methods that allowed dissecting complex chromosome rearrangements in individual cells [123]. Single-cell sequencing studies in breast cancer, investigating the evolutionary dynamics of copy number variations, found that complex aneuploidy appears early in tumor evolution and propagates by clonal expansion [124,125]. This finding contradicts the long-standing belief that complex aneuploid karyotypes develop gradually over time, but speaks to the idea that large changes in karyotype can sometimes be instantly advantageous in a specific tumor microenvironment, leading to cancer progression. Rapid and random changes in dosages of multiple genes on multiple chromosomes have a potential of giving the cancer cell the karyotype for better fitness.

### 2.9. Aneuploidy and Drug Resistance

Aneuploidy may promote the emergence of antibiotic-resistant infections and chemotherapy-resistant cancers. The budding yeast *S. cerevisiae* has traditionally been used as a model to study mechanisms of adaptations to various stresses. Yeast can rapidly adapt to unfavorable conditions by changing their karyotype. For instance, the gain of chromosome XV in budding yeast confers resistance to the antibiotic radicicol (inhibitor of chaperone protein HSP90), while the loss of chromosome XVI confers resistance to another antibiotic, tunicamycin [126,127]. Importantly, this resistance was related to the dosage of certain genes encoded on the extra chromosomes. In the case of radicicol, resistance was caused by the overexpression of two genes encoded on chromosome XV, *STI1* (co-chaperone of Hsp90) and *PDR5* (a pleiotropic drug efflux pump).

*C. albicans* and *C. neoformans*, two human fungal pathogens, frequently become aneuploid during infection and after antifungal treatments [128,129]. Drug resistance is a very common and serious problem with these pathogens. One of the first-line antibiotics for *Candida* fungal infections is fluconazole, a triazole antifungal medication that interferes with the sterol biosynthesis pathway, leading to defects in cell wall synthesis. Resistance to fluconazole has been frequently observed in clinical isolates. Sometimes this resistance can be attributed to mutations in the sterol biosynthesis pathway. However, almost half of the clinical isolates that are resistant to fluconazole carry extra copies of chromosome 5 [36]. In this case, fluconazole resistance can be narrowed down to two genes encoded on *C. albicans* chromosome 5: *ERG11* (lanosterol 14-alpha-demethylase, a component of the sterol biosynthesis pathway targeted by fluconazole), and *TAC1*, a regulator of drug efflux pumps [34].

In mammals, aneuploidy likely drives some chemotherapeutic drug resistance in cancers. Chromosomally unstable cancer cells tend to develop increased resistance to various chemotherapeutic drugs when compared to their stable counterparts [130,131,132]. The specific molecular mechanisms for drug-resistant phenotypes are not yet clearly tracked to specific genes. Prolonged exposure of human colorectal cancer cell lines to the anti-cancer drug Irinotecan led to the selection of cells containing an extra copy of chromosome 14 [133]. It was also reported that resistance to the anti-cancer drug 5-fluorouracil was increased in derivative colorectal cell lines experimentally engineered to be trisomic [47]. Findings in aneuploid rodent cell lines are consistent with the idea that multidrug resistance can occur through selection of novel aneuploidies [131,134]. Thus, as in the case of other environmental challenges, drug challenges create a novel niche to allow cells with altered chromosome constitutions selective survival and growth advantages [135].

## 3. Micronuclei

### 3.1. Footprint of Mitotic Error

Micronuclei, as an outcome of mitotic errors in higher eukaryotes, have long been observed, but only in recent years have researchers begun to pay close attention to their causes and consequences. A micronucleus is a tiny nucleus that forms from a lagging chromosome or a fragment of a chromosome that fails to incorporate into the main nucleus. When segregated sister chromatids de-condense and the nuclear envelope re-forms around them in telophase (the last stage of mitosis), spatially isolated chromosomes or chromosome fragments also de-condense, forming a small round nucleus enclosed by its own nuclear membrane (Figure 5). Micronuclei can be viewed as a footprint of chromosome missegregation that persists after mitotic exit and can be visualized in interphase cells. For pathologists, micronuclei often serve as a marker of chromosomal instability in aggressive cancers, and also as a tool to assess the genotoxicity of various chemicals [136].

### 3.2. Causes and Consequences of Chromosome Entrapment in Micronuclei

It has been reported that cytokinesis can directly generate certain structural disruptions (chromosome breakage, nuclear envelope rupture) due to entrapment of chromatin from lagging chromosomes or chromosome bridges in the cleavage furrow [100,137]. Entrapment of chromatin in the cleavage furrow appears to be a common mechanism for generating micronuclei [138]. Further DNA damage in micronuclei occurs in the subsequent interphase. The nuclear envelope around micronuclei is abnormal. Its nuclear-cytoplasmic trafficking functions are defective, and the nuclear envelope may even undergo catastrophic collapse [139,140]. The deficient nuclear-cytoplasmic trafficking prevents proper communication of the micronucleus with the rest of the cell, including propagation of the DNA damage signaling. Thus, DNA damage in micronuclei is unable to elicit a robust cellular DNA damage checkpoint response [139,140]. Failure of the DNA damage checkpoint allows cells with micronuclei containing damaged DNA to reenter mitosis. The formation of micronuclei has been linked to chromothripsis, a form of genomic instability where an individual chromosome breaks into many fragments that religate at random [141,142]. Advances in single-cell sequencing methods have allowed direct demonstration that chromosomes trapped in micronuclei can undergo chromothripsis [44]. Defective DNA replication in micronuclei has been postulated to lead to chromosome fragmentation followed by random relegation [139,143]. A recent study used inducible inactivation of the Y chromosome centromere to control the timing of micronucleus formation, demonstrating that chromothripsis in this system occurs over multiple cell cycles [144]. A chromosome within a micronucleus first undergoes defective replication or fails to repair DNA damage, then becomes fragmented as the chromosome condenses in early mitosis. When incorporated back into the parent nucleus at mitotic exit, the fragments are re-ligated at random by the DNA repair mechanisms.

## 4. Aneuploidy in Meiosis

### 4.1. Causes of Aneuploidy in Meiosis

**Figure 6:**
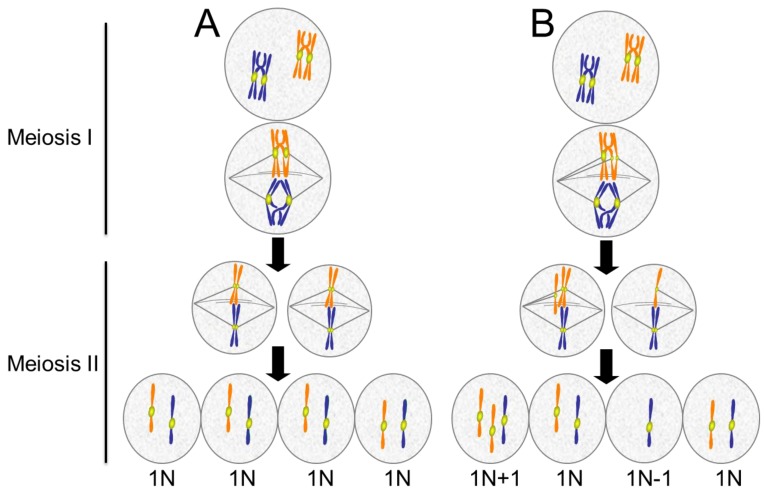
Meiotic errors lead to aneuploid gametes. (**A**) Normal meiosis consists of two chromosome segregation events without an intervening S phase. In Meiosis I, homologous chromosomes pair and undergo recombination, forming crossovers. In anaphase of Meiosis I, the homologous chromosomes segregate. In Meiosis II sister chromatids separate. The final product is four haploid (1N) cells; (**B**) Defects in meiosis result in aneuploidy. In the example shown, the chromatids of one chromosome separate prematurely and segregate to opposite poles resulting in an imbalance of chromatids in the two cells produced by Meiosis I. When these cells undergo Meiosis II, each produces one normal haploid (1N) gamete and one aneuploid (1N + 1 or 1N − 1) gamete. For simplicity, only two chromosome pairs are depicted.

Meiosis is a specialized form of cell division. It consists of two chromosome segregation events that generate haploid gametes. In humans, aneuploidy in meiosis is a major cause of infertility, miscarriage, and congenital birth defects. It is estimated that 25% to 70% of human conceptions result in aneuploid embryos, most of which are spontaneously eliminated very early in development [145]. In the first division, termed meiosis I, the homologous chromosomes pair, undergo recombination, and segregate from each other (Figure 6). Homologous chromosomes are held together through the chiasmata formed by recombination. Defects in the assembly, maintenance, or positioning of the chiasmata on the chromosomes can result in failure of homologs to orient and move to opposite poles in meiosis I [146,147]. The second division, meiosis II, is similar to mitosis, where sister chromatids segregate. Premature separation of sister chromatids, depicted in Figure 6B, is an important contributor to meiotic aneuploidy.

Most human aneuploidy results from defects during oogenesis. In males, checkpoints to detect and eliminate aneuploid cells during spermatogenesis are robust [148]. Studies in mice show that while segregation defects increase with age in germ cell division in males, checkpoint pathways successfully eliminate the cells before they mature into sperm [149]. In females, checkpoints during oogenesis appear to be weaker. The spindle checkpoint in mouse oocytes does respond by delaying or arresting cell division in the presence of several misaligned chromosomes but appears to be incapable of detecting single chromosomes that fail to align properly [150,151,152]. The significant difference between male and female meiotic events appears as a consequence of differences in the cytoplasm-to-nuclear ratio, which is much higher in oocytes. Microsurgically bisecting the cytoplasm of oocytes decreases this ratio and increases the oocyte checkpoint response to unaligned chromosomes [153]. Thus, it seems that in situations where the cytoplasm-to-chromatin ratio is very high, the checkpoint signals are too diluted to be effective. In the case of *Xenopus* eggs, which can be over 1 mm in size, the spindle checkpoint signal from chromosomes in meiosis and in early embryos is too weak to effectively block cell cycle progression, even when the spindles are completely disrupted with microtubule drugs [154]. Concentrated extracts of *Xenopus* eggs can be made that recapitulate the cell cycle in the test tube [155]. These extracts similarly lack responsiveness to microtubule inhibitors unless they are supplemented with a high concentration of sperm nuclei, a source of chromosomes. The extracts then become responsive to microtubule drugs and arrest in M phase of the cell cycle [156]. The addition of the sperm nuclei decreases the cytoplasm-to-chromatin ratio, and the combined spindle checkpoint signaling from the concentrated chromosomes becomes competent to arrest the cell cycle in M phase.

### 4.2. The Maternal Age Effect

In humans, maternal age is a major risk factor for conception of aneuploid embryos. Studies from fertility clinics show that the proportion of aneuploid oocytes increases substantially in older women [157,158]. One suspected cause is age-related decreases in the already weak spindle checkpoint signaling in oocytes. Evidence showing decreased expression of checkpoint signaling proteins or checkpoint competence in older human and mouse oocytes is consistent with this hypothesis [69,159,160,161]. Even stronger evidence suggests that a significant contributor to the maternal age effect is compromised cohesion between sister chromatids. Cohesion is mediated by the Cohesin protein complex and is released by proteolytic cleavage of one component of the complex. During meiosis, cohesion between sister chromatids is released in two stages. In anaphase of meiosis I, Cohesin on the distal parts of the chromatids is cleaved to allow separation of the homologous chromosomes that have exchanged arms by recombination. The Cohesin near the kinetochores remains protected. At the onset of anaphase in meiosis II, the Cohesin holding sister kinetochores together is cleaved, allowing chromatids to move to opposite poles.

**Figure 7:**
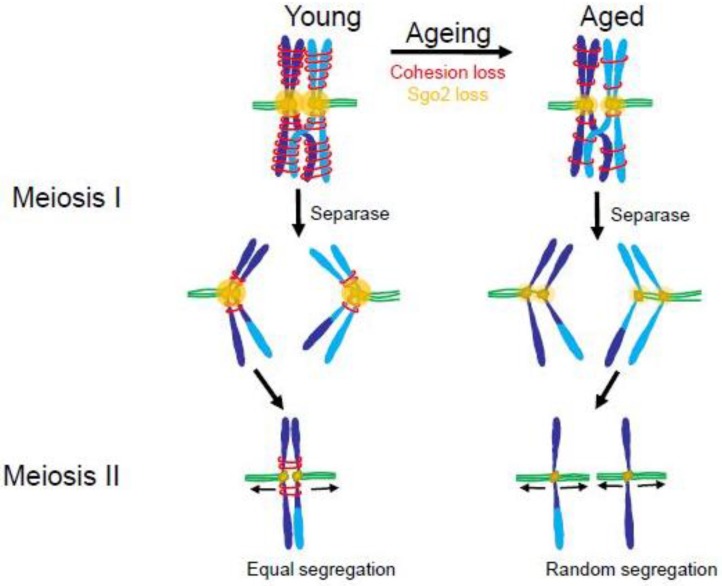
Loss of cohesin and the cohesin protector, Sgo2, in older mammals may lead to increased premature chromatid separation in meiosis. In young mammals (left) paired homologous chromosomes have high levels of cohesin and Sgo2 in Meiosis I (MI). At anaphase of Meiosis I, the protease, Separase, clips Cohesin on the distal chromosome arms allowing the homologous chromosomes to separate. Sgo2 protects Cohesin near the centromeres until Meiosis II (MII) ensuring that sister chromatids will orient to opposite poles. In aged mammals (right) there are diminished amounts of Cohesin and Sgo2. During Meiosis 1 Separase cleaves the majority of cohesin, including that near the centromeres. This allows sister chromatids to separate during anaphase of Meiosis I. In Meiosis II the individual chromatids may separate randomly to the spindle poles leading to a high incidence of aneuploidy. (Adapted from [163].)

In meiosis I, sister kinetochores show wider separation in oocytes from older women, suggesting that the Cohesin between them is compromised [162]. Elegant imaging studies of oocytes from older mice show a tendency for the kinetochores of sister chromatids to separate prematurely during anaphase of meiosis I [163]. These separated chromatids then orient randomly in meiosis II (Figure 7). Why is sister chromatid cohesion compromised in oocytes from older mammals? According to current understanding, in mammals, the Cohesin complex can only be “established”, which means made competent to hold sister chromatids together, immediately after replication in S phase [164]. In the ovaries of female mammals, S phase in oocytes occurs entirely during fetal life. Upon sexual maturity, those oocytes formed in the fetus then mature and are released over time during the female mammal’s reproductive life. Studies in mice have confirmed the idea that the Cohesin complexes established during fetal life are necessary and sufficient for chromatid cohesion in the oocytes of mature females [165,166]. Extending to humans, this system would necessitate that the Cohesin proteins established on the oocyte chromosomes during S phase in the fetus remain intact for decades. Thus, it is reasonable to suspect that such incredibly long-lived proteins might suffer some degradation in function over the years. Indeed, evidence in mouse and human suggests that Cohesin and Cohesin regulators such as the Shugoshin 2 (Sgo2) protein in mammalian oocytes decay with age, allowing sister chromatids to separate prematurely and segregate randomly in meiosis II (Figure 7) [163,167,168,169,170]. However, a recent study of chromosome distributions in human oogenesis suggests that there may exist a mysterious rescue pathway whereby chromatids that prematurely separate in meiosis I are, in some unknown way, biased to segregate to opposite poles in meiosis II, thus generating an oocyte with a normal complement of chromosomes [171].

### 4.3. Consequences of Aneuploidy in Meiosis

In mammals, whole chromosome aneuploidies on the level of the entire organism are highly detrimental for all chromosomes except sex chromosomes. Embryonic development appears to be extremely sensitive to the gene dosage imbalance caused by gains or losses of somatic chromosomes. In mice, gain or loss of any somatic chromosome is embryonic lethal. In humans, most somatic chromosome aneuploidies are also fatal. Most cases of meiotic aneuploidy result in spontaneous abortions in early pregnancy, causing more than half of miscarriages during the first trimester [172,173,174]. However, gains of certain small somatic chromosomes in humans can be viable. Viable somatic trisomies manifest in severe congenital diseases: Down syndrome (trisomy 21), Edwards syndrome (trisomy 18), and Patau syndrome (trisomy 13). Although the severity of phenotypes varies among patients, Down syndrome, caused by the gain of chromosome 21, the smallest somatic chromosome with the least number of genes, shows the highest viability. Today, the projected life expectancy of individuals with Down syndrome in developed countries is around 60 years [175], while in Edwards and Patau syndromes life expectancy is very short. Edwards and Patau syndromes are usually fatal before birth or within the first year of life, with only about 10% of patients surviving until 10 years of age.

Sex chromosome aneuploidies are viable with less severe phenotypes compared to somatic trisomies, likely because the gene dosage imbalances for genes encoded on sex chromosomes are reduced compared to genes encoded on somatic chromosomes. The absence of one sex chromosome (45,XO) causes Turner syndrome, one of the most common chromosomal abnormalities in women [176]. Since gene expression from one of the copies of X chromosomes in females is almost completely inactivated in a process called X-inactivation [177], the dosage of most X chromosome genes in XO females is comparable to normal females [178]. Turner syndrome manifests mainly in infertility that can be accompanied by heart defects and learning disabilities. Because of X inactivation, women with triple X (XXX) syndrome also have a very mild phenotype [179].

Gains of X chromosomes in males causes Klinefelter’s syndrome. Most frequently there is a gain of one extra copy of the X chromosome, resulting in the karyotype of 47,XXY. However, other karyotypes have been detected such as 48,XXXY, 48,XXYY and 49,XXXXY [180]. Because extra X chromosomes are inactivated in Klinefelter’s males in the same way as in normal females, genes on these extra X chromosomes are mostly silenced and do not cause a severe gene dosage imbalance [181]. Individuals with Klinefelter’s mainly suffer from infertility and low testosterone levels.

Y chromosome polysomies, particularly 47,XYY syndrome, also occur frequently and are frequently detected only by chance because the condition often lacks clinical manifestations. In this case, a phenotype is absent likely because the Y chromosome is very gene-poor, and genes located on the Y chromosome are not essential for viability. Therefore, increased dosage of the Y chromosome genes does not severely impair the development and physiology. Most 47,XYY males have normal sexual development and normal fertility.

### 4.4. Meiotic Aneuploidy and Cancer

Increased rates of chromosome instability lead to higher risks of certain malignancies in meiotic aneuploidy patients. For instance, Down syndrome patients have a high risk of acute myeloid leukemia, particularly the megakaryoblastic subtype [182], and trisomy 21 is frequently found in megakaryoblastic leukemia not associated with Down syndrome [183]. Human chromosome 21 contains genes encoding hematopoietic transcription factors ERG and ETS2, which are involved in megakaryopoiesis and have been shown to play a role in leukemia development. It is plausible that increased dosage of these two genes may play a role in predisposing Down syndrome patients to megakaryoblastic leukemia [184,185]. Edwards and Patau syndromes are usually fatal in the first year of life, but the few patients who survive with these trisomies for several years are predisposed to developing Wilms’ tumor, a form of kidney cancer [186]. It is not clear whether genes located on the extra chromosomes play specific roles in the development of this cancer.

## 5. Polyploidy

### 5.1. Sources for Polyploidy

There are several ways for cells to become polyploid. Most are tied to incomplete cell division. Cytokinesis normally separates the cell into two after chromosome segregation. Failure of cytokinesis produces a single tetraploid binucleate cell. In the mammalian liver, embryonic cells are primarily mononucleate but, depending on species, the adult liver contains a high percentage of binucleate cells, at least some of which are thought to occur as a result of incomplete cytokinesis [187]. Cytokinesis failure can be caused by defects in many proteins involved in this process [188,189]. It can also be blocked experimentally by chemical inhibition of the acto-myosin system of the contractile ring, which normally constricts and separates daughter cells. For instance, cytochalasins block actin polymerization. The presence of a chromosome bridge between daughter cells may cause failure to complete the process termed abscission—the final step of cytokinesis [190,191].

Failure of the spindle checkpoint to maintain mitotic arrest in cells with disrupted mitotic spindles results in cells transiting from M phase to G1 without undergoing normal mitosis and cytokinesis. This abnormal cell cycle transition is sometimes termed “mitotic slippage”. If cells exit mitosis with all chromosomes retained in one nucleus, the reformed interphase cell is tetraploid. Mitotic slippage occurs because of the gradual proteolysis of the main mitotic cyclin, Cyclin B1, which continues slowly even in checkpoint-arrested cells [192]. Cyclin B1 is the activator of the primary mitotic kinase, Cdk1. When the level of Cyclin B1 falls below a certain threshold, Cdk1 activity declines precipitously and cells exit M phase. Experimentally, mitotic slippage can be induced by treatment of cells with chemical inhibitors of Cdk1 or the spindle checkpoint kinase, Mps1 [193,194,195].

There are several instances where the generation of polyploid cells is a normal feature of differentiation. One form of this polyploidization is called endomitosis and occurs in megakaryocytes during their differentiation. Megakaryocytes are large cells with multilobed nuclei responsible for generating platelets, which mediate blood clotting. Cell cycles in megakaryocytes consists of rounds of DNA replication followed by brief entry and exit from a modified M phase. During this M phase, the nuclear envelope breaks down, chromosomes show partial condensation, and mitotic spindles form. However, the sister chromatids do not fully condense, nor do the cells undergo cytokinesis. Multiple rounds of these modified cell cycles result in massive polyploidy, a normal aspect of megakaryocyte differentiation [196,197]. Fully differentiated polyploid megakaryocytes become very large and eventually disintegrate into numerous platelets that enter circulation.

Endoreplication is another form of polyploidization where the genome replicates multiple times without intermittent entry into M phase [198]. These unusual cell cycles are called endocycles and the resulting polyploidy is called endopolyploidy. A well-known example of endoreplication is the polytene chromosomes formed in cells of the salivary glands of *Drosophila* larva. Repeated rounds of DNA synthesis without intermittent mitoses lead to the formation of multistranded chromosomes, each composed of hundreds of strands. This massive increase in DNA content increases protein production during larval development. These long, thick, multi-stranded polytene chromosomes have a characteristic appearance with easily distinguished “puffs” indicative of active transcription. Historically, polytene chromosomes may be the earliest observed banded chromosomes, paving the road for modern cytogenetics, the study of chromosome organization and function [199]. In mammals, endoreplication is an important aspect in the development of extra-embryonic tissues [200]. In trophoblast giant cells, endoreplication can reach very high ploidies, up to hundreds of folds in rodents [200]. The formation of these cells appears essential for normal embryo implantation and post-implantation functions of the placenta [201].

Another path to polyploidization, not tied to mitotic errors, is cell fusion. Cell fusion may be the source of at least some of the binucleate cells commonly present in the adult liver [202,203]. Several cancer-causing viruses, such as Hepatitis B and C, Epstein-Barr, and Human Papilloma Virus (HPV) are also fusogenic [204]. Unlike polyploidy which stems from mitotic errors or endoreplication, cells of different types can fuse and produce hybrid polyploid cells. Such hybrids may gain characteristics from both original cell types. For instance, the fusion of cancer cells with bone marrow–derived cells can produce malignant hybrid cancer cells with traits from bone marrow that may promote metastasis [205].

### 5.2. Polyploidy in Fungi

In many lower eukaryotes, the genome displays a high degree of plasticity in terms of both polyploidy and aneuploidy. Some fungi can proliferate as either haploid or diploid. Various types of fungi can also become polyploid through failed cell divisions, at least in laboratory settings. Subsequently, they propagate as polyploid or transition to being aneuploid by losing some of the extra chromosomes during subsequent cell divisions. Polyploidy, whole chromosome aneuploidy, and segmental aneuploidy are very common in domesticated populations of *S. cerevisiae* beer yeasts [24]. Laboratory strains of *S. cerevisiae* can be propagated at ploidies up to pentaploid. In certain conditions, polyploidization in yeast is adaptive. The encapsulated yeast *C. neoformans*, an opportunistic fungal pathogen, can cause pneumonia and meningitis in immunocompromised individuals. It can generate large polyploid cells called “titan cells”. These cells are most frequently tetraploid or octoploid and are resistant to the host immune system and to the antifungal antibiotic fluconazole partly because they have a thicker cell wall and a sturdier, denser capsule [37,206]. Another opportunistically pathogenic yeast, *C. albicans,* routinely inhabits skin and mucous membranes. In immunodeficient individuals it can generate infections that, after exposure to fluconazole and other azole antifungals, can harbor tetraploid cells [207]. These tetraploid cells generate abnormal mitotic spindles in subsequent cell cycles, leading to aneuploidy in the progeny.

### 5.3. Polyploidy in Animals and Plants

Most species of the animal kingdom, with few exceptions, are diploid, and polyploidy of whole animals is unusual. In mammals, only one species is known to carry extra sets of chromosomes: the red viscacha rat, *Tympanoctomys barrerae* [208]. A few hundred cases of polyploid species are known among insects, reptiles, amphibians, crustaceans and fish [209]. Polyploidy can play a role in the evolution of animal species. One example is the commonly used lab species *Xenopus laevis*, the South African clawed toad. This frog appears to have evolved from the mating of two closely related species whose offspring retained both sets of chromosomes [210]. This condition is called allotetraploidy. Millions of years of evolution have generated many genetic changes, including deletions of duplicate genes, but a recent genome evolution study revealed that the species retains active alleles from both ancestors in over 56% of genes [211]. Chance genome doubling during mammalian embryogenesis typically leads to embryonic lethality. In humans, congenital triploidy and tetraploidy may account for up to 10% of spontaneous abortions [212]. Interestingly, a few case studies report live births of complete and mosaic tetraploid humans with severe developmental defects [213,214,215].

In stark contrast to the situation in animals, polyploidy is very common in plants, especially in angiosperms (flowering plants) [216]. The exact reasons for this tolerance are unknown, but it may be at least partially due to the absence of p53 in plants [217]. Polyploid plants are typically bigger than their diploid ancestors with correspondingly larger fruits. It is estimated that up to 30% of wild angiosperms are polyploid, and more than 50% of angiosperms that comprise agricultural and food crops can have ploidies ranging from triploid to octoploid and beyond [218]. Most agricultural crops are angiosperms. Polyploidy is very prominent in the grass family—the source of wheat, rice and corn. Other crops including potatoes, sugarcane, apples, strawberries, bananas, coffee are also polyploid [219]. This polyploidy is a product of human selection for plants with bigger, bulkier fruits, and is largely responsible for feeding modern humanity.

### 5.4. Polyploidy Can Lead to Aneuploidy

**Figure 8:**
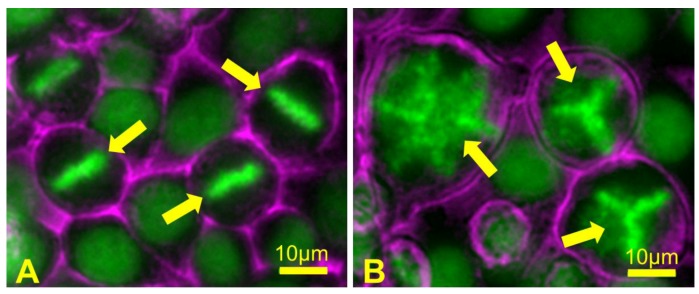
Failed cytokinesis can lead to multipolar spindle formation in the subsequent mitosis. (**A**) A field of control HeLa cells containing mitotic cells showing normal chromosome alignment at metaphase (arrows); (**B**) HeLa cells were treated for a short time with an actin polymerization inhibitor drug, which blocks cytokinesis and results in the formation of binucleate polyploid cells containing extra centrosomes. During the subsequent mitosis, polyploid cells form abnormal metaphase chromosome alignments (arrows) when the presence of extra centrosomes leads to assembly of multipolar mitotic spindles. When these cells then undergo anaphase and cytokinesis, chromosomes are segregated in a random, unequal manner, leading to the formation of daughter cells that are highly aneuploid. Chromosomes are depicted in green; cell surfaces are depicted in magenta.

Polyploid animal cells are prone to chromosome missegregation during mitosis [220]. If polyploid cells contain supernumerary centrosomes, then cell divisions in these cells can be error-prone, because multipolar mitotic spindles that often form when there are more than two centrosomes cannot segregate sister chromatids equally (Figure 8). However, cells with extra centrosomes can and do generate bipolar mitotic spindles by clustering centrosomes to form two spindle poles [221]. Even with bipolar spindles, such cells still display an increased tendency for chromosome missegregation during anaphase [222].

An example of a normal tissue that progresses from polyploidy to aneuploidy is the adult mammalian liver where, in addition to the binucleate cells containing two diploid nuclei, binucleate and mononucleate cells polyploid and aneuploid cells are also present [223]. The percentage of binucleate cells declines after partial hepatectomy and regrowth, giving rise to both polyploid and aneuploid cells through abnormal divisions [223]. Notably, the existence of stable polyploid and aneuploid cells in normal mammalian liver without massive induction of cancer suggests that ploidy alterations on their own are not sufficient for oncogenesis, at least in hepatocytes.

As mentioned earlier, aneuploidy in fungi can be a consequence of polyploidy induced in response to anti-fungal agents. In the laboratory, tetraploid *S. cerevisiae* strains have a 200- to 1000-fold increase in chromosome loss rates relative to diploid cells, due, at least in part, to a higher incidence of syntelic kinetochore attachments, where both sister chromatids attach to the same spindle pole [224]. Polyploid titan cells in *C. neoformans* lung infections can rapidly produce diverse aneuploid progeny, which promotes adaptation to antibiotic therapy [37]. Similarly, in opportunistically pathogenic *C. albicans*, tetraploid cells generated after exposure to antifungal agents can produce aneuploid progeny that acquire antibiotic resistance [207].

### 5.5. Polyploidy in Cancer

Oncogenesis is a multi-step evolutionary progression that selects for traits that allow malignant cells to survive and proliferate. With the advances in whole genome sequencing technology, it has become possible to trace cancer genome changes during oncogenic progression. Tetraploidization appears to be a relatively common event in tumor evolution. Evidence for transient genome-doubling events has been detected during progression of malignancies, and tetraploidization has been associated with cancer aggressiveness and recurrence [225,226,227,228]. Tetraploidy may be an early event in tumorigenesis that fuels further genomic instability, leading to the selection of tumors with increased malignancy [229,230,231,232]. This idea has been tested in mice using a xenograft cancer model that began with non-malignant diploid or tetraploid xenograft cells. This approach demonstrated that tetraploid cells were tumorigenic when injected in immunodeficient mice and gave rise to malignant aneuploid cells, while isogenic diploid cells did not [233,234,235]. The aneuploid cells of tumors derived from tetraploid precursors demonstrated a wide diversity of karyotypes indicating a high degree of chromosomal instability.

Polyploid cells often contain extra centrosomes, and their cell divisions can be catastrophically error-prone because, as indicated previously, cells with extra centrosomes are prone to assemble multipolar mitotic spindles. Since multipolar spindles cannot segregate chromosomes equally, essentially all progeny become aneuploid. These aneuploid cells may also exhibit chromosome instability and acquire further numerical and segmental chromosomal aberrations [220,222,236,237,238]. However, it is also common that in cancer cells, multiple centrosomes can cluster to form bipolar spindles [239]. Strategies to inhibit centrosome clustering and thus purposefully drive spindle multipolarity have been proposed as potential cancer therapies [240,241]. Indeed, it has been proposed that the well-established anti-cancer drug Taxol may function in this manner [87]. On the other hand, in human tumors, the presence of abnormal mitotic figures such as multipolar spindles in biopsies is considered a feature of advanced malignancy. Understanding the positive and negative consequences of spindle multipolarity remains an important topic for future study.

Tetraploid cells in tissue culture can also reveal increased resistance to certain chemotherapeutic drugs compared to their parental diploid cells [242,243,244,245,246]. This effect is reminiscent of the elevated antibiotic resistance detected in polyploid fungi, although the mechanisms underlying this resistance in mammalian cells have not been discovered.

## 6. Ploidy Aberrations and P53

### 6.1. Ideas in Evolution and Cancer

In mammals, aneuploidy- and polyploidy-driven evolution of single cells is restrained by the tumor suppressor protein p53 [2,3,5,233,247,248]. p53 is a transcription factor that regulates the expression of various genes involved in stress responses, cell cycle arrest, and apoptosis. Yeast cells do not have the p53 gene, and homologues of mammalian p53 first appeared in protostomes (molluscs, annelids and arthropods) [249]. As mentioned previously, plants also lack p53 [217]. Animals with a large body size require many more cells and often exhibit longer lifespans than smaller animals. Thus, long-lived, large animals might be expected to have an increased susceptibility to cancer. However, no correlation between body size or lifespan and the occurrence of cancer can be found [250,251,252]. Interestingly, elephants possess 20 copies of the p53 gene and show a hyperactive p53-dependent DNA damage response, potentially contributing to cancer resistance in this large, long-lived animal [253].

More than half of human malignancies harbor mutations of the p53 gene [254], and together with alterations in other components of the p53 network, the p53 pathway is suppressed or inactivated in most human cancers [255]. Inactivation of the p53 pathway likely unleashes cancer evolution, enabling cancer cells with abnormal karyotypes to proliferate, limited only by their fitness in a given environment.

### 6.2. Concepts for Activation and Function

p53 is one of the most extensively studied proteins, yet it is still not clear what specific factor, or combination of factors, triggers its activation in cells with aberrant ploidies. Determining this has been challenging because transcriptional activation of p53 can be triggered by a wide variety of external and internal stresses [256,257,258], and the range of stresses that may occur in polyploid and aneuploid cells is also broad. The roles of p53 in sensing DNA damage as well as oxidative and proteotoxic stresses are well established. These stressors can accompany some cases of ploidy alterations. In addition, the stoichiometry of ribosomal proteins caused by changes in gene dosages can activate the p53 pathway by protecting p53 from ubiquitination by its key negative regulator, the ubiquitin ligase Mdm2, which targets it for degradation by the proteasome [259]. Moreover, polyploid and aneuploid cells frequently have an aberrant number of centrosomes, and recent studies show that the p53 may be activated by extra or missing centrosomes [260,261,262]. A recent study of chromosome missegregation in anaphase demonstrated that lagging or misaligned chromosomes stabilize p53 through retained phosphorylation of histone H3.3, suggesting that mitotic defects resulting in missegregated chromosomes can activate p53 directly [247].

When non-transformed cells in culture are induced to become polyploid by disruption of the actin or microtubule cytoskeleton, they usually block proliferation through the expression of p53 [2,233,263,264,265,266,267,268]. Direct imaging of cells in culture suggests that the p53-dependent arrest may be delayed for up to three cell cycles after the induction of polyploidy [269]. The pathways linking polyploid cell formation to p53 activation remain unclear. One mechanism may be activation of the Hippo tumor suppressor pathway, induced, at least in part, by extra centrosomes [262]. In tissue culture, proliferating lines of tetraploid cells can grow out from cultures experimentally induced to become tetraploid. These karyotypically stable tetraploid cells adapt to contain normal numbers of centrosomes and build bipolar mitotic spindles [43,262]. As yet, a full understanding of how aneuploid and polyploid cells circumvent p53-mediated arrest remains incomplete, but two recent studies indicated that overexpression of D-type Cyclins allows the continued proliferation of tetraploid cells despite the presence of wild-type p53 [43,270].

In a variety of cell lines, loss of function mutations of the Rb tumor suppressor caused significant chromosome segregation errors but only a modest increase in aneuploidy, unless p53 was also inactivated, whereupon aneuploidy was greatly enhanced [271]. In cells, heterozygous for an inactivating p53 mutation, loss of Rb function could increase the probability that a segregation error causes loss of the chromosome containing the wild-type p53 allele (loss of heterozygosity) and thus generate proliferative progeny permissive for further chromosome instability and increased aneuploidy. Of note, aneuploid cells also occur naturally in some tissues such as the adult liver [223] and the brain [272]. As discussed earlier, polyploidization is a part of a normal differentiation program in certain cell lineages. In tissue culture, human pluripotent stem cells and RPE-1 with normal p53 expression were found to gain extra copies of chromosome 12 and proliferate at a high rate [43]. It is not yet fully clear how, in various circumstances, acquisition of extra chromosomes in non-malignant p53-expressing cells in culture allows cells to evade detection or overcome activation of p53.

## 7. Conclusions and Perspectives for Human Health

The simple use of the terms mitotic or meiotic *errors* presupposes that such events are detrimental. In many, perhaps most cases, abrupt changes in chromosome content in humans will have unfavorable consequences, for example meiotic aneuploidies giving rise to abnormal embryos or cancer cells developing increased malignancy. However, studies in unicellular eukaryotes have demonstrated that aneuploidy and genomic instability can empower adaptive evolution. Here, there are distinct differences between single-cell eukaryotes and metazoans. In yeast, karyotypic diversity is limited only by the fitness cost and may allow exploitation of new environmental conditions. In multicellular organisms, proliferative competition of individual cells leads to cancer and compromises the fitness of the whole organism. However, there are clear instances where altered mitotic events have been subsumed into differentiation, providing evolutionary advantages in metazoans.

The study of the paths leading from segregation errors to adverse consequences has important potential for human health. For example, in individual cancers, what is the relationship among chromosome instability, aneuploidy, and malignancy? Which pathways—sister chromatid cohesion, cell cycle checkpoints, chromosome movement, and others—are affected? Can we take advantage of cancer’s dependence on a specific auxiliary mitotic pathway to design therapies that are generally nontoxic to normal cells but lethal to the tumor? Can we design treatments that specifically target cells that are aneuploid? Can prevention of centrosome clustering be a viable cancer therapy? After many years of study, the complex role of p53 in tumor progression still holds secrets. How can these be revealed and exploited? What are the most important contributors of the maternal age effect? Is it possible to design interventions that might promote normal fertility and development? As we learn more about the mechanisms underlying mitosis and meiosis, we are sure to uncover more surprising insights into the complex interplay of the regulation of cell division with disease, health, and evolution.

## Figures and Tables

**Table 1:** Definitions.

**Ploidy** is the number of sets of chromosomes in a cell or in an organism.
**Haploid** number refers to one set of chromosomes (1N), as in gametes or certain strains of budding yeast.
**Diploid** number refers to two sets of chromosomes (2N) that are homologous (one from each parent). Most animals are diploid.
**Polyploid** denotes a cell with more than two sets of chromosomes (triploid – 3N, tetraploid – 4N, pentaploid – 5N, etc.).
**Euploid** denotes the normal chromosome number in a species, usually an exact multiple of the haploid number (i.e.; human euploid genome contains 46 chromosomes – 2× the haploid number).
**Chromosomal Instability** is the tendency of a cell to gain or lose chromosomes or large segments of chromosomes. It is often abbreviated CIN.
**Aneuploidy** denotes the state of a cell having a chromosome number that deviates from a multiple of the haploid, i.e.; when there are extra or missing single chromosomes.
**Whole chromosomal aneuploidy** is having entire chromosomes gained or lost.
**Segmental aneuploidy** is having large regions of chromosomes deleted, duplicated or translocated from one chromosome to another. Cancer cells often exhibit both whole chromosome aneuploidy and segmental aneuploidy.
**Trisomy** refers to a diploid genome having gained an additional chromosome (2N + 1). Trisomy 21 indicates an extra chromosome 21 in a diploid genome.
